# Novel *MNX1* mutations and genotype–phenotype analysis of patients with Currarino syndrome

**DOI:** 10.1186/s13023-020-01442-4

**Published:** 2020-06-22

**Authors:** Lu Han, Zhen Zhang, Hui Wang, Hui Song, Qing Gao, Yuchun Yan, Ran Tao, Ping Xiao, Long Li, Qian Jiang, Qi Li

**Affiliations:** 1 Department of Medical Genetics, Capital Institute of Pediatrics, No. 2 Yabao Rd., Chaoyang District, Beijing, 100020 China; 2 Department of General Surgery, Capital Institute of Pediatrics Affiliated Children’s Hospital, No. 2 Yabao Rd., Chaoyang District, Beijing, 100020 China; 3 Department of Radiology, Capital Institute of Pediatrics Affiliated Children’s Hospital, Beijing, 100020 China; 4 Department of Pathology, Capital Institute of Pediatrics Affiliated Children’s Hospital, Beijing, 100020 China

**Keywords:** Currarino syndrome, *MNX1*, Genotype–phenotype analysis, Recurrent, Noncanonical splice site variant

## Abstract

**Background:**

Currarino syndrome (CS) is a specific complex of congenital caudal anomalies, including anorectal malformations, presacral mass and sacral anomalies. Mutations in the *MNX1* gene are closely related to CS and occur in almost all familial cases and less than half of sporadic patients. We investigated the spectrum of *MNX1* pathogenic variants and associated clinical features in Chinese patients with CS.

**Results:**

Seventeen index patients from 16 families were recruited from 2015 to 2018. All patients were diagnosed with CS and treated at the Capital Institute of Pediatrics Affiliated Children’s Hospital. Genetic testing was applied to identify mutations in CS patients and their relatives by whole-exome sequencing and Sanger sequencing. Functional verification was performed for a recurrent noncanonical splice site variant in *MNX1* with a minigene splicing assay. In 17 CS patients, 14 were complete CS and 3 were mild CS. Nine variants in *MNX1* were identified in 11 patients, and these included two frameshift mutations (p.Leu223Leufs*61, p.X402Serfs*70), four nonsense mutations (p.Gly42X, p.Cys88X, p.Gln24X, p.Cys241X), one missense mutation (p.Trp288Leu), one splice region variant (c.691 + 3G > T) and one polyalanine polymorphism (p.Ala135insAlaAla). Seven of these nine variants have never been reported. Pathogenic *MNX1* mutations were found in 100% (4/4) of familial and 46% (6/13) of sporadic patients.

**Conclusion:**

Our study expanded the mutation spectrum of *MNX1* and provided clinical and genetic analyses of seventeen CS patients from mainland China.

## Introduction

Currarino syndrome (CS, OMIM 176450), also called the Currarino triad, was first mentioned by Currarino et al. in 1981 as a specific complex of congenital caudal anomalies, including anorectal malformations, presacral mass and partial sacral agenesis [1]. The prevalence of CS is 1 ~ 9/100,000, as reported by the Orphanet website (https://www.orpha.net). In general, CS can be divided into three categories, according to the severity of the disease: (1) complete CS: anorectal malformations (ARMs), presacral mass and sacral anomalies; (2) mild CS: sacral anomaly associated with one of the other two malformations, i.e., ARMs or presacral mass; and (3) minimal CS: only have sacral anomalies [2, 3]. Patients are classified as familial patients if characteristics of the CS phenotype are also observed in family members. The other affected individuals, in contrast, have no family history of CS and are classified as sporadic patients.

In recent decades, genetic studies have obtained clear evidence of autosomal dominant inheritance in CS, which exhibits broad expressivity and incomplete penetrance [3–5]. The detection rate of *MNX1*, the distribution of the main pathogenic variants, was different in familial and sporadic patients. Most familial patients had causative mutations in *MNX1*, while 70% of sporadic patients do not have specific related genes [2]. Thus, it is vital to accurately distinguish between familial and sporadic patients in the clinic. The diagnosis of CS is dependent on radiologic examination, in which a pelvic X-ray is used to detect sacral defects in patients with severe constipation of unknown reasons or anal malformations. Small presacral masses and associated intravertebral anomalies are sometimes overlooked and can be detected by an MRI evaluation of the pelvis and spine [6–8].

The motor neuron and pancreas homeobox 1 (*MNX1*, Gene ID 3110) gene, located on chromosome 7q36.3, encodes a nuclear protein that is highly expressed in the colon, small intestine, duodenum, pancreas and testis in humans [9]. Structurally, the protein contains a homeodomain, a highly conserved region of 82 amino acids, and a polyalanine region consisting of 16 alanines [10]. Most of the previous studies have only focused on the proband and lacked familial genetic studies. In addition, differences in mutation carrying rates among patients of different ethnic groups have been detected [2, 11]. Here, we collected a large number of Chinese patients with CS for whole-exome sequencing and Sanger sequencing to investigate the spectrum of *MNX1* pathogenic variants and associated clinical features. We characterized novel mutations that enlarge the list of the mutations identified to date and are considered causative of CS. In addition, we tried to define the functional consequence of a recurrent noncanonical splice site variant in *MNX1* with a minigene splicing assay. Finally, we reported a high frequency of parental-inherited pathogenic *MNX1* mutations in “sporadic” patients and presented extreme phenotypic variability in individuals carrying the same mutation.

## Subject and methods

### Study subjects

Seventeen index patients from 16 families were recruited in this study from January 2015 to December 2018. All patients were diagnosed with Currarino syndrome and treated at the Department of General Surgery, Capital Institute of Pediatrics Affiliated Children’s Hospital. Medical records were collected to obtain clinical information. All patients underwent X-ray examination of the sacrococcygeal region and MRI examination of lumbosacral and pelvis before operation. Pathological examinations were performed to identify the nature of the presacral mass. Of the seventeen patients, two were male and fifteen were female, ranging in age from 2 months to 6 years, with a median age of 1 year. We obtained peripheral blood samples from all 17 CS patients and 43 direct relatives to conduct a genetic analysis.

### Genetic analysis

#### Whole-exome sequencing (WES)

WES was performed in two batches on 9 families in the first round of the study. A total of 3 μg of DNA from 9 CS patients and 25 direct relatives were sent to NovelBio Biotechnology Co., Ltd. (Shanghai, China). for exome capture and sequencing. Then, we performed WES on the other 3 families in the second round of the study. 2 μg of DNA from 4 CS patients and 7 direct relatives were sent to WuXi NextCODE Co., Ltd. (Shanghai, China). for exome capture and sequencing.

Briefly, exome capture was performed with the SureSelect Human All Exon v5 Plus Library (Agilent Technologies), according to the manufacturer’s instructions (Illumina, San Diego, CA). Appropriate amounts of enrichment DNA libraries were sequenced on a HiSeq 2000 (Illumina) with 100 bp paired-end reads. Quality control (QC) filters were applied to remove reads with low quality and obtain clean data. Bioinformatics analysis was performed using an in-house pipeline that included an alignment (human reference genome hg19, NCBI) with the Burrows-Wheeler Aligner (BWA-MEM). Next, using GATK (v2.4–9), the initial BAM files were realigned, and base quality scores were recalibrated. After marking the duplicates with Picard (v1.74), the final set of alignment data (BAM files) was generated, which was used for the single nucleotide variant (SNV) and copy number variant (CNV) prediction programs. For SNV calling, we used the publicly available ‘best practices’ GATK call set made with the latest UnifiedGenotyper and a call set made with the HaplotypeCaller, using default arguments. Detected SNVs were then annotated using ANNOVAR. Further analyses were performed on frameshift mutations, in-frame indels, start/stop codon changes, missense variants, and splice region variants with a minor allele frequency < 0.5% in the 1000 Genomes Project Database (The 1000 Genomes Project Consortium 2014) and gnomAD browser (https://gnomad.broadinstitute.org/). For CNV calling, SAMtools was used to calculate the every-coding-region total bases. GATK “DepthofCoverage” command was used to obtain average mean depth of the CCDS (Consensus Coding Sequence) regions. R was then used to calculate the ratio of every sample compared with other samples` mean ratio and ggplot was used to plot the result. A ratio > 1.4 was assigned duplication and < 0.6 was assigned deletion. To reduce false positives, only deletions or duplications of two consecutive exons are identified as true variants.

#### Sanger sequencing validation and *MNX1* screening

In the first round of the study, Sanger sequencing was performed on families with suspected pathogenic mutations in *MNX1* for validation after WES. In the second round of the study, Sanger sequencing was utilized for screening *MNX1* mutations in 4 CS patients and their parents if applicable. Genomic DNA of peripheral blood leukocytes was extracted using the salt-precipitation method. One hundred and fifty nanograms of DNA were added into the PCR mixture, which contained 25 μl of 2 × GC buffer II (5 mM Mg^2+^ Plus), 5.5 μl of dNTP mixture (2.5 mM each), 2 μl of each primer working solution (20 μM), and 0.5 μl of Takara LA Taq (Takara RR02AG) to a final volume of 50 μl. Amplification was performed using PCR System 9700 (Applied Biosystem) with the following protocol: denaturation at 94 °C for 1 min, followed by 35 cycles (94 °C for 30 s, 58 °C for 30 s, and 72 °C for 2 min) and a final elongation step at 72 °C for 5 min. The result was analyzed on an ABI 3730 analyzer (Applied Biosystem). We identified sequence variants by CodonCode Aligner (v 8.1.0.1) based on the reference sequence of *MNX1* (NM_005515.3). Primer sequences are available if requested.

### Bioinformatic analysis

The potential pathogenicity of all the missense variants was predicted by the in silico analysis using four different tools: Polyphen-2 (http://genetics.bwh.harvard.edu/pph2), MutationTaster (http://www.mutationtaster.org/), SIFT (http://sift.bii.a-star.edu.sg/) [12–16] and combined annotation-dependent depletion (CADD, https://cadd.gs.washington.edu/). The potential effect on splicing was predicted with two splice-site prediction programs, Human Splicing Finder (HSF, http://www.umd.be/HSF3/HSF.shtml) and Splice Site Prediction by Network (http://www.fruitfly.org/seq_tools/splice.html). The latest American College of Medical Genetics guidelines were used to classify all the variants we detected (http://acmg.cbgc.org.cn/) [17].

### Functional study on a splice region variant in MNX1

We performed functional validation of a noncanonical splice site variant (c.691 + 3G > T) in *MNX1* with a minigene splicing assay. Amplicons generated by standard overlapping PCR and digestion procedures were cloned into two sets of vectors (pEGFP-C1-MNX1 and pcMINI-N-MNX1). PCR and Sanger sequencing were applied to evaluate whether the wild-type (wt) and mutant (mut) expression vectors had been successfully constructed. The recombinant vectors (pEGFP-C1-MNX1-wt/pEGFP-C1-MNX1-mut and pcMINI-N-MNX1-wt/pcMINI-N-MNX1-mut) were then transiently transfected into human embryonic kidney cells (HEK-293 T) and cervical cancer cells (HeLa), according to the instructions, and the transfected cells were cultured for 36 h before being collected for the analysis. Total RNA was extracted from both HEK-293 T and HeLa cells by the TRIzol method (RNA extraction kit, Omega), and cDNA was reverse transcribed using a reverse transcription kit (Thermo), according to the manufacturer’s instructions. The concentration and purity of the extracted RNA were determined by UV spectrophotometry. PCR products were identified by 2% agarose gel electrophoresis and verified by sequencing. The primers used in the experiments are shown in Supplementary Table S1. The construction of recombinant plasmids is shown in Fig. 3a and Supplementary Figure S1A.

## Results

### Clinical features of patients with CS

In this study, we included seventeen patients with a clinical suspicion of CS from 16 families. Among them, fourteen patients presented with the complete CS form (14/17, 82%), three showed two (sacral anomaly + ARM) out of three major characteristic signs and were diagnosed with mild CS (patients 2, 7 and 14). In our series, four patients (patients 5, 6, 7 and 8) had documented familial history of CS with one or more relatives showing complete triad or mild forms, and the other thirteen were classified as sporadic patients (Table 1). The sacral defect was present as the minimum inclusion criterion in 100% of the patients. Partial sacrococcygeal agenesis (type III) was the predominant form (5/17), followed by hemisacrum (type IV) in 3/17 and coccygeal agenesis (type V) in 1/17. Detailed information was not available in 8 patients, so an accurate classification could not be determined. All seventeen patients (100%) showed anorectal malformations (ARMs), ranging between anorectal stenosis (10/17), rectal perineal fistula in 5/17 and rectovestibular fistula (2/17). A presacral mass was identified in 14 of 17 patients and mainly consisted of teratoma (10/17), followed by cystic formation in 2 out of 17 patients and hamartoma and lipoma in 1 out of 17 patients each. Other signs in our CS patients included tethered cord, myelomeningocele, meningocele, spinal arachnoid cysts, and right kidney transposition/malrotation (Table 1).

**Table 1:** Clinical information of patients with Currarino syndrome

Family No.	Case No.	Sex	Age^**a**^	Sacral anomaly	Anorectal malformation (ARM)	Presacral mass	Other signs	Familial/ sporadic case^**b**^
1	1	F	1 yr	Sacral agenesis (NA)	Anorectal stenosis	Teratoma	–	S
2	2	F	3 yr	Sacral agenesis (NA)	Rectal perineal fistula	–	–	S
3	3	F	3 m	Sacral agenesis (NA)	Rectovestibular fistula	Teratoma	–	S
4	4	F	6 yr	Sacral agenesis (NA)	Anorectal stenosis	Teratoma	–	S
5	5	M	1 yr	Sacral agenesis (NA)	Anorectal stenosis	Cystic formation	–	F
6	6	F	5 yr	Coccygeal agenesis (type V)	Anorectal stenosis	Teratoma	–	F
	7	F	21 m	Partial sacrococcygeal agenesis (type III)	Rectal perineal fistula	–	Tethered cord, Myelomeningocele	F
7	8	F	3 yr	Partial sacrococcygeal agenesis (type III)	Rectal perineal fistula	Cystic formation	Tethered cord	F
8	9	F	2 yr	Hemisacrum (type IV)	Anorectal stenosis	Teratoma	Meningocele, Tethered cord	S
9	10	F	21 m	Sacral agenesis (NA)	Rectovestibular fistula	Teratoma	–	S
10	11	M	9 m	Partial sacrococcygeal agenesis (type III)	Rectal perineal fistula	Teratoma	–	S
11	12	F	2 m	Sacral agenesis (NA)	Rectal perineal fistula	Hamartoma	Right kidney transposition and malrotation	S
12	13	F	8 m	Partial sacrococcygeal agenesis (type III)	Anorectal stenosis	Teratoma	Meningocele, Tethered cord	S
13	14	F	9 m	Hemisacrum (type IV)	Anorectal stenosis	–	Tethered cord	S
14	15	F	2 yr	Partial sacrococcygeal agenesis (type III)	Anorectal stenosis	Teratoma	Spinal arachnoid cysts	S
15	16	F	10 m	Hemisacrum (type IV)	Anorectal stenosis	Teratoma	–	S
16	17	F	9 m	Sacral agenesis (NA)	Anorectal stenosis	Lipoma	–	S

^a^Age is expressed in years (yr) or months (m). ^b^F, familial case; S, sporadic case

Abbreviations: *F* female, *M* male, − absent, *NA* detailed information not available

Females were more frequently affected than males in our study. Both male patients (patients 5 and 11) had complete CS, while twelve out of fifteen female patients had complete CS. Genital malformations occurred only in female CS patients. The father of patient 5 presented with sacral anomaly, ARM and presacral mass (discovered by MRI). Patients 6 and 7 are sisters born to nonconsanguineous parents. Their father was clinically unaffected, but an MRI detected a presacral mass and sacral anomaly (undetermined type). In the large family of patient 8, 4 individuals were also affected with either the complete or mild form of CS (Fig. 2).

### Genetic findings

**Fig. 1:**
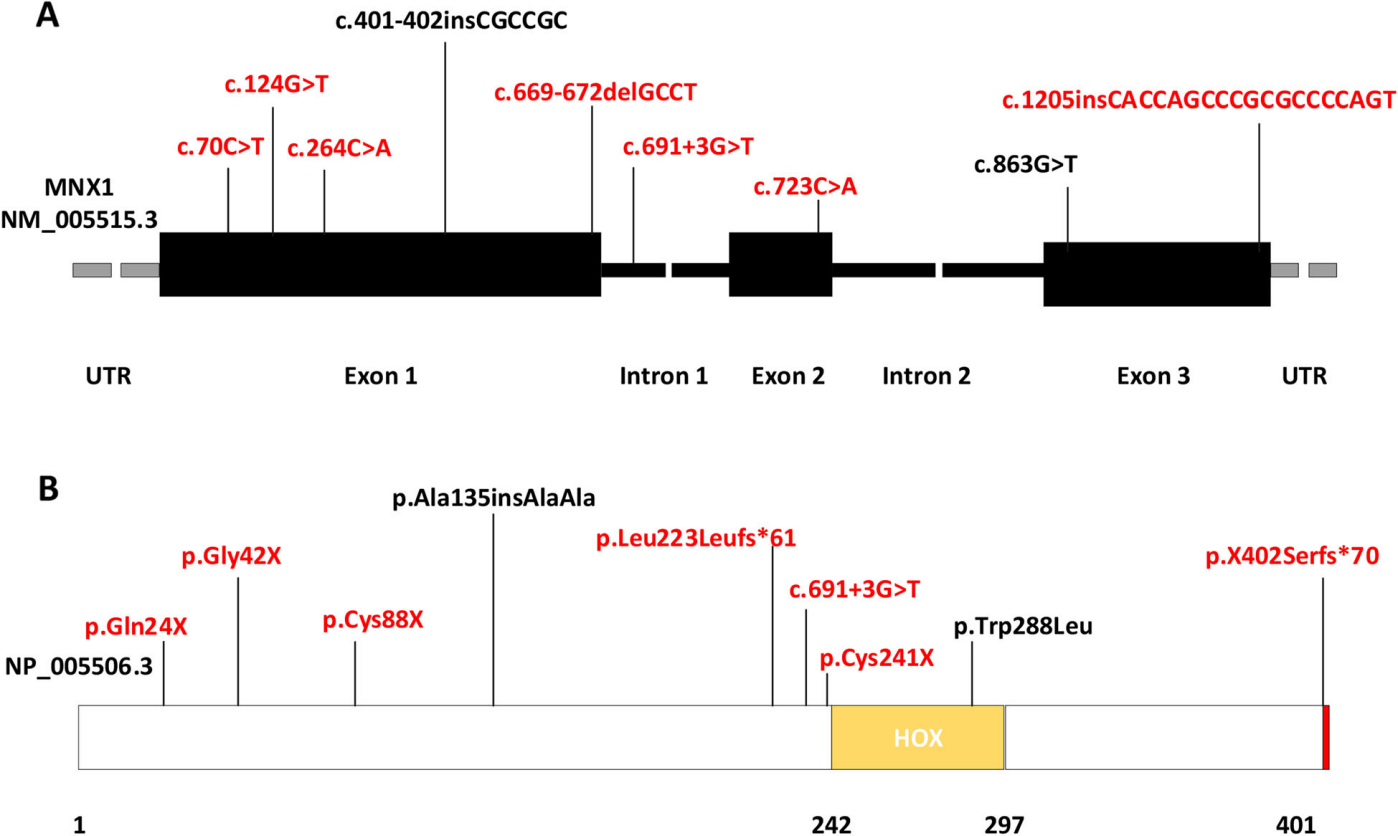
Distribution of the nine *MNX1* variants detected in CS patients in this study. **a** Schematic representation of the exon-intron structure of *MNX1*. Black bars represent exons, and black lines represent introns, with patient variants indicated above the *MNX1* genomic structure. **b** Domain structure of MNX1 (GenBank: NP_005506.3), including the positions (numbers) of identified amino acid alterations. Abbreviation: HOX, homeodomain. Novel and previously reported variants are shown in red and black, respectively, in (**a**) and (**b**)

WES was used to sequence 45 individuals (13 patients and 32 relatives) across 12 CS families. The average coverage of each sample in the exome sequencing data for the *MNX1* gene coding sequence ranged between 48.63 and 76.94 (median 66.35). Sanger sequencing was applied to screen *MNX1* in 4 patients and their parents, if applicable. In total, variants in *MNX1* were identified in 11 patients (11/17, 65%), including two frameshift mutations (p.Leu223Leufs*61, p.X402Serfs*70), four nonsense mutations (p.Gly42X, p.Cys88X, p.Gln24X, p.Cys241X), one missense mutation (p.Trp288Leu), one splice region variant (c.691 + 3G > T) and one polyalanine polymorphism (p.Ala135insAlaAla). The distribution of the nine different *MNX1* variants is shown in Fig. 1. Seven of these nine variants have never been reported (Table 2). Five out of the nine variants are located in exon 1; one is located in intron 1, one in exon 2, and two in exon 3. A highly conserved homeodomain between codons 242 and 297 was affected by the only missense mutation. According to the latest ACMG guidelines, pathogenic (or likely pathogenic) *MNX1* mutations were found in 100% (4/4) of familial and 46% (6/13) of sporadic patients (Table 2).

**Table 2:** Genetic findings in patients with Currarino syndrome

Case No.	Gene	Exon No.	Nucleotide change	Amino acid change	Mutation type	Inheritance	Reported	ACMG classification	Evidence of pathogenicity
1	*MNX1*	1	c.124G > T	p.Gly42X	Nonsense	Maternal	No	Pathogenic	PVS1 PM2 PP4
2	*MNX1*	1	c.669-672delGCCT	p.Leu223Leufs^a^61	Frameshift	Maternal	No	Pathogenic	PVS1 PM2 PP4
3	*MNX1*	1	c.264C > A	p.Cys88X	Nonsense	de novo	No	Pathogenic	PVS1 PS2 PM2 PP4
4	*MNX1*	1	c.401-402insCGCCGC	p.Ala135insAlaAla	Inframe insertion	NA	Yes	Uncertain significance	PM6 BP6
5^a^	*MNX1*	3	c.1205insCACCAGCCCGCGCCCCAGT	p.X402Serfs^a^70	Frameshift	Paternal	No	Pathogenic	PVS1 PM2 PP1 PP4
6^a^	*MNX1*	1	c.70C > T	p.Gln24X	Nonsense	Paternal	No	Pathogenic	PVS1 PM2 PP1 PP4
7^a^	*MNX1*	1	c.70C > T	p.Gln24X	Nonsense	Paternal	No	Pathogenic	PVS1 PM2 PP1 PP4
8^a^	*MNX1*	Intron 1	c.691 + 3G > T	–	Splice region mutation	Maternal	No	Likely pathogenic	PS3 PM2 PP1 PP4
9	*MNX1*	3	c.863G > T	p.Trp288Leu	Missense	Paternal	Yes	Likely pathogenic	PS1 PM2 PP3
10	*MNX1*	2	c.723C > A	p.Cys241X	Nonsense	Maternal	No	Pathogenic	PVS1 PM2 PP1 PP4
11	*MNX1*	Intron 1	c.691 + 3G > T	–	Splice region mutation	de novo	No	Pathogenic	PS2 PS3 PM2 PP4
12	*TLE4*	17	c.1949C > T	p.Ser650Leu	Missense	de novo	No	Likely pathogenic	PS2 PM2 PP3 PP4
	*HOXB4*	1	c.48G > T	p.Lys16Asn	Missense	de novo	No	Likely pathogenic	PS2 PM2 PP3 PP4
13	*ITIH2*	13	c.1623_1626delAGAG	p.Ile541Ilefs^a^12	Frameshift	de novo	0.00003252 in gnomAD	Likely pathogenic	PS2 PM2 PP4
14	*CDH2*	3	c.453G > T	p.Arg151Ser	Missense	de novo	No	Likely pathogenic	PS2 PM2 PP3 PP4

^a^Familial case

Abbreviation: *NA* not available

In addition, likely pathogenic variants were discovered in three CS patients in 4 new candidate genes. Two de novo missense variants were detected by WES in patient 12, a female sporadic CS patient: *TLE4* (p.Ser650Leu) and *HOXB4* (p.Lys16Asn). Sporadic female patient 13 was detected to carry a de novo frameshift mutation in *ITIH2* (p.Ile541Ilefs*12). In patient 14, a de novo missense variant was discovered in *CDH2* (p.Arg151Ser) (Table 2). No suspected variants were detected in patients 15, 16 and 17 by WES.

**Fig. 2:**
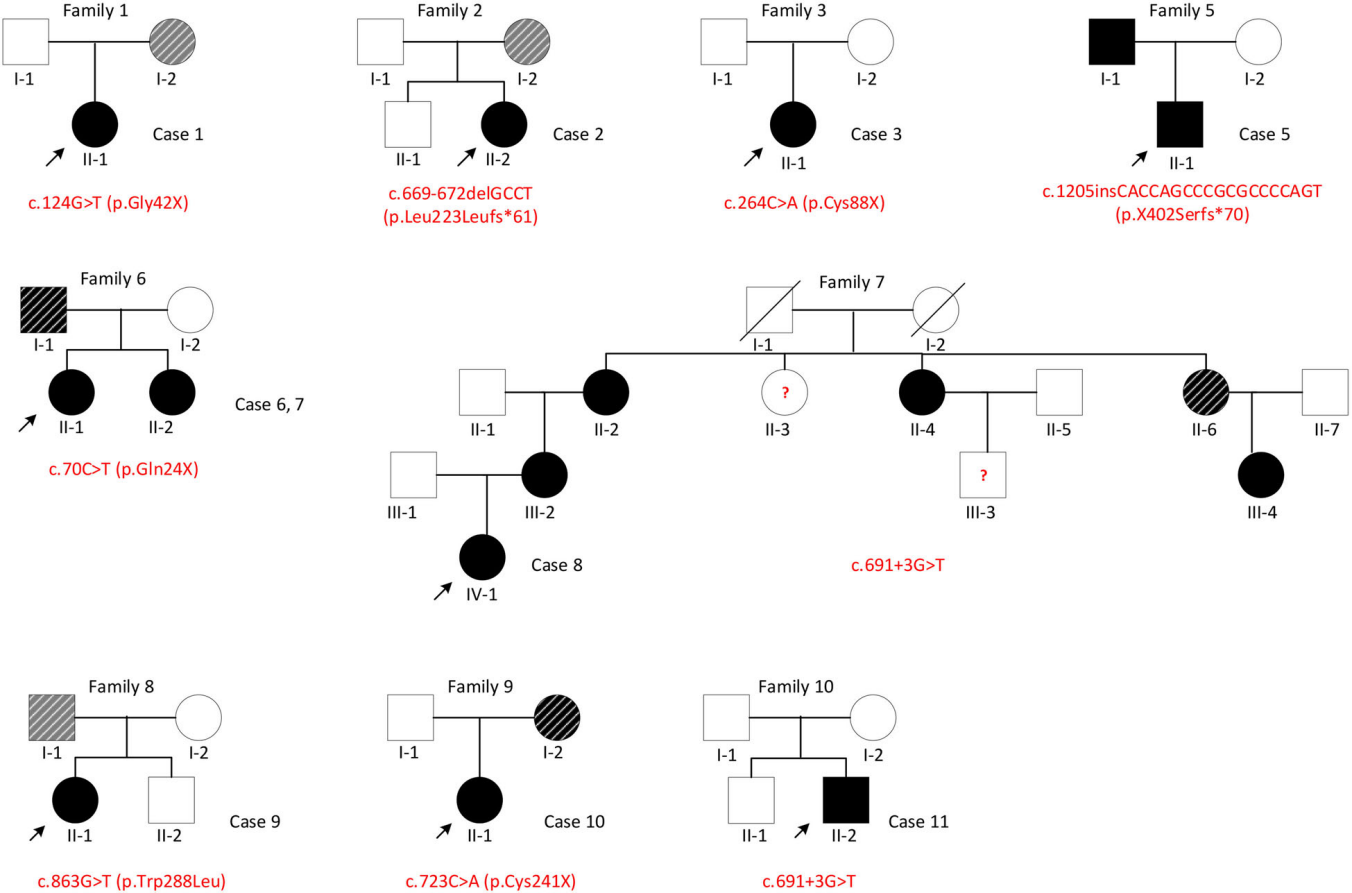
Pedigrees of the 9 families detected to carry pathogenic *MNX1* mutations in this study. Individuals with CS are indicated by solid squares (male) or solid circles (female). Affected individuals with *MNX1* mutations who have changes apparent only on an MRI are indicated by a black diagonal stripe. Asymptomatic individuals who have *MNX1* mutations are indicated by a gray diagonal stripe

Combining two sequencing methods, suspected disease-causing mutations were found in 12 families (12/16). Among these, mutations in *MNX1* were found in 9 of them (9/12). Among the seventeen CS patients and eight affected relatives (I-1 in family 5; I-1 in family 6; II-2, II-4, II-6, III-2 and III-4 in family 7; and I-2 in family 9) who had at least MRI changes, eighteen were detected to have pathogenic (or likely pathogenic) variants in *MNX1* (18/25). These include six sporadic CS patients (the proband in family 1 to 3 and family 8 to 10) and four familial patients. Of the six sporadic patients, all of their parents and siblings also had their genes sequenced. Four (4/6, 66.7%) inherited the *MNX1* mutation from one of their parents (3 maternal and 1 paternal), and the other two (2/6, 33.3%) had de novo mutations. In the familial patients, the *MNX1* mutation was paternally inherited in three patients (patient 5 in family 5; patients 6 and 7 in family 6) and maternally inherited in one patient (patient 8 in family 7). The phenotypes and family structures of those 10 CS patients carrying pathogenic *MNX1* mutations are depicted in Fig. 2.

### Minigene study on the noncanonical splice site variant in MNX1

Notably, the noncanonical splice site variant (c.691 + 3G > T) in *MNX1* was detected in two unrelated families, patient 8 in family 7 and patient 11 in family 10. As shown in Fig. 2, multiple family members of patient 8 were also diagnosed with CS (II-2, II-4, III-2 and III-4) or discovered to have a presacral mass by MRI (II-6), and all carried the same variant, indicating a strong co-segregation. Functional assays were then performed to determine the effect of the variant on mRNA splicing.

**Fig. 3:**
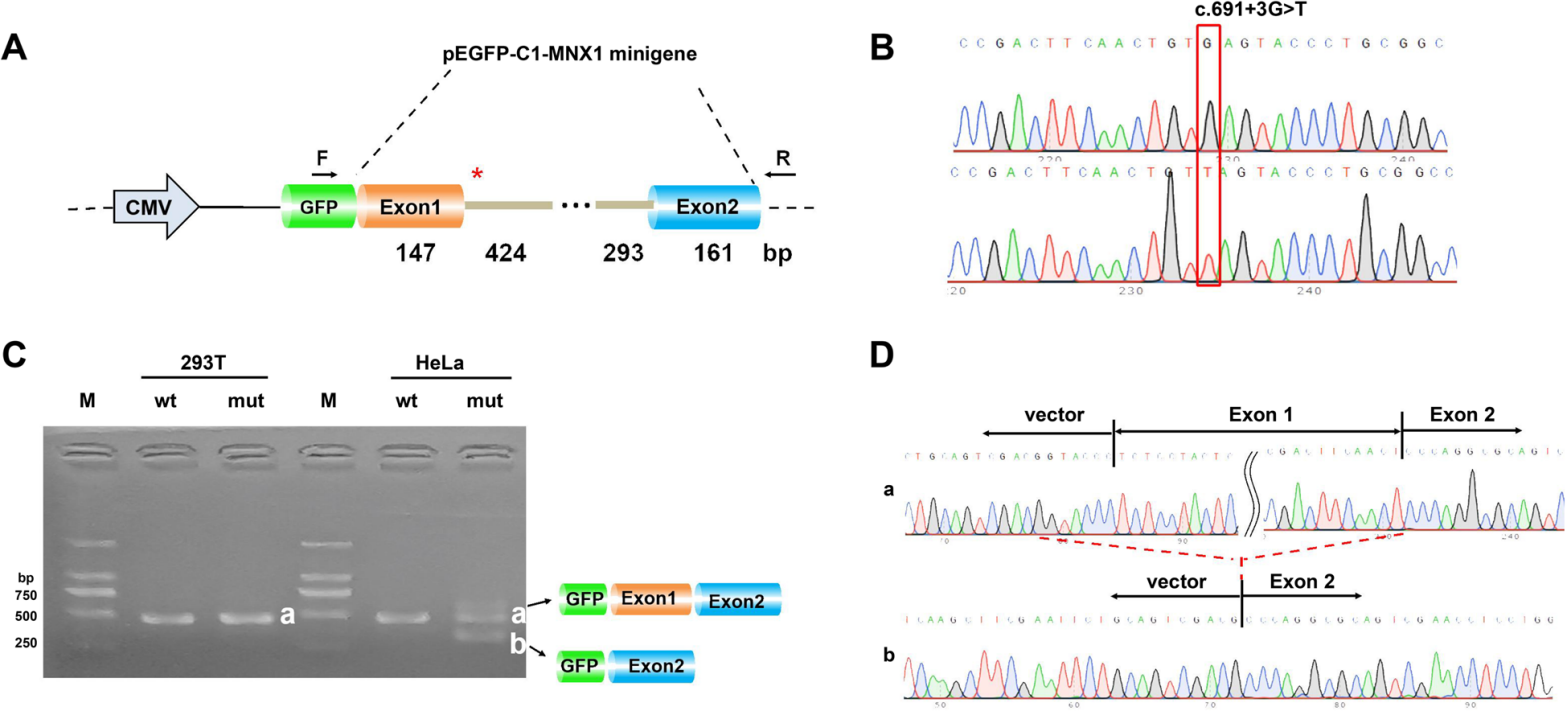
Minigene study on a recurrent noncanonical splice site variant in ***MNX1*. a** Structure of the splicing vector pEGFP-C1 and minigene MNX1-wt/MNX1-mut (c.691 + 3G > T): the pEGFP-C1 vector contains a CMV promoter, and the symbol “*” represents the location of the mutation. **b** Sequencing results of the target fragment with wild-type (wt) at the top and mutant (mut) at the bottom. **c** Reverse-transcription polymerase chain reaction (RT-PCR) products were separated by electrophoresis in HEK-293 T (left) and HeLa (right) cells. **d** Minigene product sequencing results: a, the wild-type minigene (MNX1-wt) formed normal mRNA composed of exons 1 and 2; b, the mutant intron c.691 + 3G > T minigene caused a splicing abnormality in HeLa cells, resulting in the skipping of the 147 bp base in exon 1

In the study with the pEGFP-C1 vector, the RT-PCR results showed that there was only one wt (wild-type) and one mut (mutant) band in 293 T cells, both with the expected size and were named band a. In contrast, the mutant plasmid transfection in HeLa cells produced two bands, one with the expected size that was named band a, and the other was an obviously smaller size and named band b (Fig. 3c). Sequencing results showed that the wild-type band a was cleaved normally, i.e., the cleavage mode of the band was GFP-exon 1 (147 bp) and -exon 2 (161 bp) (Fig. 3d-a). Mutant band b skipped the entire exon 1 with a cleavage mode similar to GFP-exon 2 (161 bp) (Fig. 3d-b). The RT-PCR sequencing results with vector pcMINI-N are shown in Supplementary Figure S1; mut produced the same sized band as wt in both 293 T and HeLa cells. These results show that the *MNX1* mutant minigenes may produce alternative transcripts different from the wt minigene in certain cell types.

### Genotype–phenotype analysis

In our collections of seventeen CS patients, we identified 9 different *MNX1* mutations; of these mutations, 7 were novel and 2 had been described previously. All variants were found in a heterozygous state. Among the novel mutations, 3 were detected in familial patients: (1) p.X402Serfs*70 was found in a boy with a complete CS phenotype, and his father was affected by sacral anomaly, ARM and presacral mass (discovered by MRI); (2) p.Gln24X was present in a female with complete CS consisting of coccygeal agenesis, anorectal stenosis and teratoma; her younger sister also carried the nonsense mutation and presented mild CS with partial sacrococcygeal agenesis and rectal perineal fistula. MRI screening in the clinically unaffected father detected a presacral mass and sacral anomaly of an undetermined type; (3) for a noncanonical splice site variant c.691 + 3G > T, wide intrafamilial clinical variability was observed, ranging by a complete CS triade with association of partial sacrococcygeal agenesis, rectal perineal fistula and cystic formation in the proband (IV-1 in family 7) to an asymptomatic isolated constipation in the grandaunt (II-6). Moreover, in a sporadic male patient (II-2 in family 10) carrying the same variant, partial sacrococcygeal agenesis, rectal perineal fistula and teratoma were observed as a complete form of CS. The other four novel mutations were all discovered in sporadic female patients: three mutations (p.Gly42X, p.Cys88X and p.Cys241X) were detected in patients with complete CS, and one (p.Leu223Leufs*61) was detected in a patient with mild CS. The only missense mutation discovered in our study was p.Trp288Leu, and it has been described multiple times in patients with complete CS [10, 18]. A female patient (in family 8) in the current study was affected by hemisacrum, anorectal stenosis and teratoma. She inherited the mutation from her father who had no clinical symptoms and refused an MRI test.

To search for a correlation between the nature or position of the mutation and the severity of the exhibited phenotypes, we considered the patients carrying mutations that are all distributed in the coding region but did not find any genotype-phenotype correlations. Additionally, from the comparison of the patients with complete CS (14/17, Table 1), we could not identify a correlation with the genotype because they all indistinctly carried different mutation types (nonsense, frameshift, splicing and missense mutations).

## Discussion

Here, we offered a set of genetic research data with a large sample size in mainland China and reported 7 novel *MNX1* mutations that are all pathogenic and considered causative of CS. In our series, we observed a higher mutation detection rate in familial forms of the disease, with a mutation present in 4 of 4 familial patients (100%) compared to 6 of 13 sporadic patients (46%). Furthermore, when the mutation was inherited, it was prevalently transmitted through the paternal line (3 of 4) in familial patients and through the maternal line in sporadic patients (3 of 4). The mutation detection rate in our sporadic patients is higher than that reported by others: 25% (6/24) in South Korea [4], 33% (6/18) in Italy [2], and 7% (1/14) in Norway [19]. One possible explanation is that we may have underestimated the familial forms due to not being able to perform a radiologic examination of the sacral region in some parents (i.e., I-2 in family 1 and I-1 in family 8) who were apparently healthy but resulting carriers of an *MNX1* mutation. In this view, *MNX1* genetic testing played an essential role from the clinical reevaluation of misdiagnosed patients for the purpose of a more accurate familial recurrence risk calculation [20].

Among the seventeen CS patients and eight affected relatives who had at least MRI changes, only eighteen were detected to have pathogenic (or likely pathogenic) variants in *MNX1*. On the other hand, by comparing patients with complete CS, we could not identify a correlation with the genotype because they all indistinctly carried different mutation types (nonsense, frameshift, splicing and missense mutations). Finally, wide clinical variability was observed in all our intrafamilial patients, i.e., ranging from a complete CS triade in the proband of family 7 to an asymptomatic isolated constipation in her grandaunt. That is, a very detailed clinical investigation of all patients failed to identify any obvious genotype-phenotype correlations, similar to previous investigations [4, 18]. Although the expression of *MNX1* is limited by haploinsufficiency in Currarino syndrome, the degree of transcriptional repression of the nonmutated allele may not be identical for every patient with a given mutation, and this could explain the large intra-mutational phenotypic variability. Alternatively, modifier genes have been suggested to participate in the formation of different phenotypes [21–23].

The distribution of previously described *MNX1* mutations suggests mutational predilection sites of between positions 125 and 130, a stretch of six cytosine residues, and between positions 408 and 413, a stretch of six guanine residues, and these might be caused by the replication slippage mechanism [11, 18]. However, variants detected in the current study were scattered throughout the whole coding sequence of *MNX1*. For p.Gln24X, p.Gly42X, p.Cys88X, p.Leu223Leufs*61 and p.Cys241X, since alleles with frameshift or nonsense mutations in the N-terminus of the homeobox encode proteins that lack the homeodomain, they represent loss-of-function alleles, leading to *MNX1* haploinsufficiency. A noncanonical splice site variant (c.691 + 3G > T) was discovered in two unrelated families and showed strong co-segregation in one large pedigree. We then performed a minigene splicing assay to test its functional consequence. The RT-PCR sequencing results indicate that this mutation may cause exon 1 skipping in the HeLa cell line and thus disturb mRNA splicing. The mutation in patient 9 has previously been described in independent patients of different ethnic origins and with different clinical and phenotypic characteristics [10, 18]. All bioinformatics analyses indicated that W288L is probably damaging to the protein function. Polyalanine repeats are an increasingly recognized motif at the N-terminal side of both homeodomain proteins and in nonhomeodomain transcription factors and might be associated with transcriptional repressor activity [24]. Indeed, mutations have been reported within the region encoding the polyalanine repeat in *MNX1*, and variation in the length is considered to be associated with the presence of Currarino syndrome [11, 25].

In addition, likely pathogenic variants were discovered in 4 new candidate genes in three sporadic CS patients in this study. *TLE4,* encoding a transcriptional corepressor, has a broad expression in the testis, bone marrow and 20 other tissues [9]. *HOXB4* is a member of the Antp homeobox family and encodes a nuclear protein with a homeobox DNA-binding domain. The encoded protein functions as a sequence-specific transcription factor that is involved in development [9, 26]. The protein encoded by *ITIH2* belongs to the inter-alpha-trypsin inhibitor (ITI) family, members of which are structurally related plasma serine protease inhibitors involved in the stabilization of the extracellular matrix and in the prevention of tumor metastasis [27]. *CDH2*, encoding a classical cadherin and member of the cadherin superfamily [9], plays a role in the establishment of left-right asymmetry, development of the nervous system and the formation of cartilage and bone [28].

## Conclusion

Genetic analysis of *MNX1* is a necessary step in a CS diagnostic protocol to identify those subjects who are apparently asymptomatic and is a useful approach to better distinguish between familial and sporadic patients in the clinic. In view of the absence of a clear genotype-phenotype correlation and broad phenotypic expression, it is difficult to predict the outcome severity in newborns or small children when performing a diagnostic test. However, an early diagnosis is still strongly advisable for the follow-up and treatment options for severe complications of CS and for the a more accurate familial recurrence risk calculation and genetic counseling.

## Supplementary information

**Additional file 1: Table S1.** Primers used in the minigene splicing assay. **Figure S1.** Minigene study on a recurrent noncanonical splice site variant in *MNX1*. (A) Structure of the splicing vector pcM1N1-N and minigene MNX1-wt/MNX1-mut (c.691 + 3G > T): the pcM1N1-N vector contains a T7 promoter, and the symbol “*” represents the location of the mutation. (B) Sequencing results of the target fragment with wild-type (wt) at the top and mutant (mut) at the bottom. (C) Reverse-transcription polymerase chain reaction (RT-PCR) products were separated by electrophoresis in HEK-293 T (left) and HeLa (right) cells. (D) Minigene product sequencing results: a, both the wild-type minigene (MNX1-wt) and the mutant (mut) minigene (MNX1-mut) formed normal mRNA composed of exons 1 and B.

## Data Availability

All data generated or analysed during this study are included in this published article and its supplementary information files.

## Abbreviations

CS: Currarino syndrome
*MNX1*: Motor neuron and pancreas homeobox 1
ARMs: Anorectal malformations
WES: Whole-exome sequencing
QC: Quality control
SNV: Single nucleotide variant
CNV: Copy number variant
CCDS: Consensus Coding Sequence
WT: Wild-type
Mut: Mutant
