# Supplementary material for: Novel MNX1 mutations and genotype–phenotype analysis of patients with Currarino syndrome
Source: Orphanet J Rare Dis. 2020 Jun 22;15:155. doi: 10.1186/s13023-020-01442-4 (PMC7310025; doi:10.1186/s13023-020-01442-4)

**Supplementary Material**

Novel *MNX1* mutations and genotype–phenotype analysis of patients with Currarino syndrome

Lu Han, Zhen Zhang, Hui Wang, Hui Song, Qing Gao, Yuchun Yan, Ran Tao, Ping Xiao, Long Li, Qian Jiang, Qi Li

Correspondence to: Dr. Qian Jiang at the Department of Medical Genetics, Capital Institute of Pediatrics, No. 2 Yabao Rd., Chaoyang District, Beijing 100020, China (teaco@126.com); and Dr. Qi Li at the Department of General Surgery, Capital Institute of Pediatrics Affiliated Children's Hospital, No. 2 Yabao Rd., Chaoyang District, Beijing 100020, China (l817@sina.com)

**Supplementary Table S1. Primers used in the minigene splicing assay**

| **Name** | **Sequence** |
| --- | --- |
| pcMINI-N -MNX1-Kpn1- F | GCTTGGTACCATGCTCTCCTACTCGTACCCGC |
| pcMINI-N -MNX1-BamH1-R | TAGTGGATCCgcgggtctctctaacgccccag |
| MNX1-MUT-F | GCCCGACTTCAACTgtTagtaccctgcggcc |
| MNX1-MUT-R | ggccgcagggtactaacAGTTGAAGTCGGGC |
| GFP-MNX1-kpn1-new-F | CGACGGTACCcTCTCCTACTCGTACCCGCAGGTG |
| GFP-MNX1-BamH1-R | CGGTGGATCCCTGGGTCTCGGTGAGCATGAG |
| GFP-MNX1-F | cgttagagagacccgcgtggagaggaagcctc |
| GFP-MNX1 -R | gaggcttcctctccacgcgggtctctctaacg |

**Supplementary Figure S1. Minigene study on a recurrent noncanonical splice site variant in *MNX1*.** (A) Structure of the splicing vector pcM1N1-N and minigene MNX1-wt/MNX1-mut (c.691+3G>T): the pcM1N1-N vector contains a T7 promoter, and the symbol “*” represents the location of the mutation. (B) Sequencing results of the target fragment with wild-type (wt) at the top and mutant (mut) at the bottom. (C) Reverse-transcription polymerase chain reaction (RT-PCR) products were separated by electrophoresis in HEK-293T (left) and HeLa (right) cells. (D) Minigene product sequencing results: a, both the wild-type minigene (MNX1-wt) and the mutant (mut) minigene (MNX1-mut) formed normal mRNA composed of exons 1 and B.


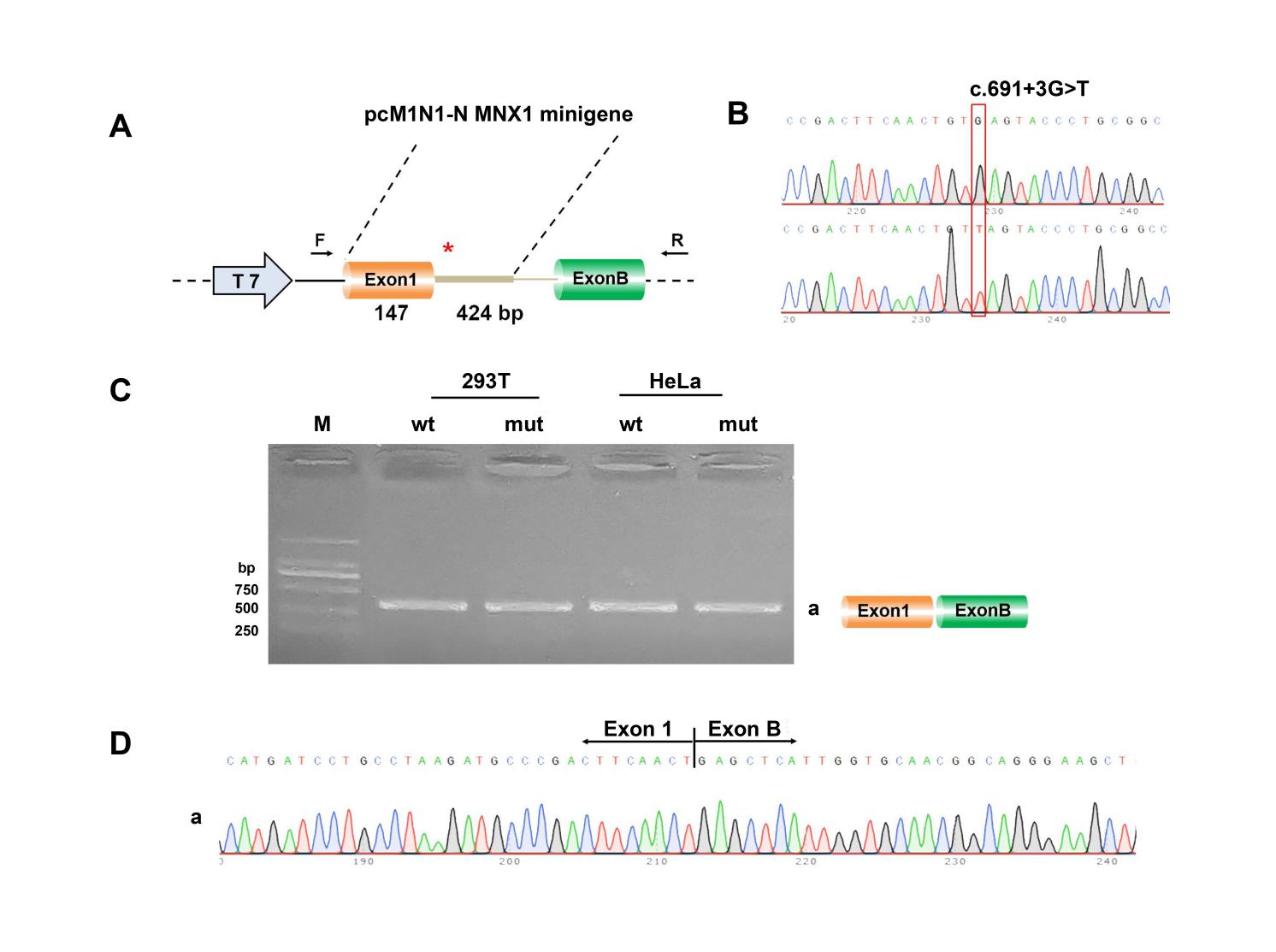

Supplement: Supplementary file 1 — Additional file 1: Table S1. Primers used in the minigene splicing assay. Figure S1. Minigene study on a recurrent noncanonical splice site variant in MNX1. (A) Structure of the splicing vector pcM1N1-N and minigene MNX1-wt/MNX1-mut (c.691 + 3G > T): the pcM1N1-N vector contains a T7 promoter, and the symbol “*” represents the location of the mutation. (B) Sequencing results of the target fragment with wild-type (wt) at the top and mutant (mut) at the bottom. (C) Reverse-transcription polymerase chain reaction (RT-PCR) products were separated by electrophoresis in HEK-293 T (left) and HeLa (right) cells. (D) Minigene product sequencing results: a, both the wild-type minigene (MNX1-wt) and the mutant (mut) minigene (MNX1-mut) formed normal mRNA composed of exons 1 and B. [file 13023_2020_1442_MOESM1_ESM.docx]
